# Difference of gut microbiota between patients with negative and positive HBeAg in chronic hepatitis B and the effect of tenofovir alafenamide on intestinal flora

**DOI:** 10.3389/fmicb.2023.1232180

**Published:** 2023-09-20

**Authors:** Jianfei Long, Jingru Gong, Han Zhu, Xiaolin Liu, Ling Li, Bicui Chen, Hongyan Ren, Chao Liu, Huiping Lu, Jiming Zhang, Bin Wang

**Affiliations:** ^1^Department of Pharmacy, Huashan Hospital, Fudan University, Shanghai, China; ^2^Department of Pharmacy, Shanghai Pudong Hospital, Fudan University Pudong Medical Center, Shanghai, China; ^3^Department of Pharmacy, Jing’an District Central Hospital, Fudan University, Shanghai, China; ^4^Shanghai Mobio Biomedical Technology Co., Ltd., Shanghai, China; ^5^Department of Infectious Diseases, Shanghai Key Laboratory of Infectious Diseases and Biosafety Emergency Response, National Medical Center for Infectious Diseases, Huashan Hospital, Fudan University, Shanghai, China; ^6^Shanghai Institute of Infectious Diseases and Biosecurity, Key Laboratory of Medical Molecular Virology (MOE/MOH), Shanghai Medical College, Fudan University, Shanghai, China; ^7^Department of Infectious Diseases, Jing’An Branch of Huashan Hospital, Fudan University, Shanghai, China

**Keywords:** hepatitis B virus, HBeAg, HBsAg, tenofovir alafenamide, gut microbiota

## Abstract

**Background:**

Severe liver diseases, such as liver fibrosis, cirrhosis, and liver cancer, are mainly caused by hepatitis B virus (HBV). This study investigated the differences between gut microbiota in HBeAg-positive and negative groups of patients with chronic hepatitis B (CHB) and investigated the effect of tenofovir alafenamide (TAF) on gut microbiota.

**Methods:**

This prospective study included patients with CHB not taking nucleoside antivirals (No-NAs group, *n* = 95) and those taking TAF (TAF group, *n* = 60). We divided CHB patients into two groups according to the HBeAg status of the subjects on the day of data collection. Phase 1 are HBeAg-negative patients and phase 2 are HBeAg-positive patients. We investigated the improvement of clinical symptoms by TAF, as well as differences in gut microbiota between different groups by 16S rRNA high-throughput sequencing.

**Results:**

Gut microbiota demonstrated significant differences between patients with HBeAg-positive and -negative CHB. Both the No-NAs and TAF Phase 2 subgroups demonstrated significantly increased microbiota richness and diversity, showing greater heterogeneity. Additionally, the Phase 2 subgroup exhibited a low abundance of pathways associated with glucose metabolism and amino acid metabolism. The TAF group demonstrated a significantly decreased HBV load, alanine aminotransferase, and aspartate aminotransferase and a significant increase in prealbumin compared with the No-NAs group. No significant difference was found in uric acid, creatinine, blood calcium, inorganic phosphorus, eGFR, and β2-microglobulin concentrations between the two groups. Additionally, the urea level in the TAF group was significantly lower than that in the No-NAs group, but with no significant effect on other indicators such as eGFR and β2-microglobulin.

**Conclusion:**

This study revealed significant differences in gut microbiota composition and function between patients with HBeAg-positive and -negative CHB.

## 1. Introduction

Hepatitis B virus (HBV) infection is a global public health problem, and patients with chronic hepatitis B (CHB) frequently experience persistent low-grade liver inflammation (Lavanchy, 2004; Ott et al., 2012). HBV may cause severe liver diseases, such as liver fibrosis, cirrhosis, and liver cancer (Ding et al., 2012; Zeng et al., 2021). The World Health Organization reported that approximately 296 million people worldwide are infected with HBV (Chinese Society of Hepatology Chinese Medical Association; Chinese Society of Gastroenterology Chinese Medical Association; Chinese Society of Infectious Diseases, Chinese Medical Association, 2022). The liver is the initial organ that contacts substances from the gut, and nutrients, bacterial metabolites, or toxins from the gut enter the liver and play an important role in liver disease progression (Wiest et al., 2017). Increasing evidence indicated the important role of gut microbiota in liver disease development, progression, and treatment response (Schnabl and Brenner, 2014; Wang et al., 2017; Liu et al., 2019). Patients infected with HBV have altered intestinal permeability, increased bacterial and endotoxin translocation, and promoted immune-mediated liver injury (Kassa et al., 2021). Microorganism and bacterial toxin translocation, such as lipopolysaccharides, have been reported to exacerbate the clinical features of chronic liver disease (Woodhouse et al., 2018).

HBV infection cumulatively affects gut microbiota (Yun et al., 2019; Zhu et al., 2019). HBV is a hepatotropic virus, and HBV e-antigen (HBeAg) is a soluble secreted form of HBcAg and a viral replication serological marker. Hepatitis B seroconversion is classified into (1) HBeAg-negative and e antibody positive, called HBeAg seroconversion; or (2) HBsAg negative and s antibody positive, called HBsAg seroconversion. Both seroconversions suggest host immune control and low HBV replication (Bonino et al., 2010). However, studies on gut microbiota between patients with HBeAg-positive and -negative CHB have not been reported. HBeAg development may be associated with gut microbiota, as HBeAg clearance has been induced in HBeAg-positive patients by fecal bacterial transplantation, and symptom improvement in these patients is accompanied by significant changes in the gut microbiota composition (Ren et al., 2017). Additionally, HBeAg in patients with CHB can reduce TLR2 expression in hepatic Kupffer cells and monocytes (Kawasaki and Kawai, 2014). Thus, the effect of HBeAg on the gut microbiome profile remains to be investigated to identify potential microbiome targets to mitigate HBV infection. Additionally, investigating the differences in gut microbiota between HBeAg -positive and -negative infected individuals contribute to our understanding of HBV pathogenesis.

Tenofovir is currently the first-line treatment for HBV infection. Tenofovir is available in two main drug forms: tenofovir disoproxil fumarate (TDF) and tenofovir alafenamide (TAF). TAF is characterized by lower plasma pK exposure, lower nephrotoxicity, and less impact on bone structural integrity, thus TAF has gradually emerged as the drug of choice for HBV treatment (Di Perri, 2021; Kumada et al., 2021). Studies have demonstrated that entecavir can improve the intestinal flora of patients with CHB (Lu et al., 2021), but the effect of TAF on the gut microbiota has not been reported. Further investigation of the effect of TAF on gut microbiota can better understand the link between gut changes, considering its smaller amount and smaller toxicity, and their effect on the hepatic immune response is essential for improving HBV treatment.

Hence, our study investigated the differences in gut microbiota between 95 patients with CHB receiving no nucleoside analog drugs and 60 patients with CHB receiving TAF by high-throughput 16S rRNA sequencing, as well as the characteristics of gut microbiota in HBeAg-positive and -negative patients in different groups. This study aimed (1) to investigate the changes in the structure and diversity of the microbial community in HBeAg-positive and -negative patients during HBV infection, (2) to determine the effect of TAF on the gut microbiota of patients with HBV and the differences in metabolic pathways associated with it, and (3) to explore the correlation between gut microbiota and clinical parameters.

## 2. Methods

### 2.1. Study subjects

This study was conducted at Huashan Hospital from January 2020 to December 2021 and recruited 95 patients with CHB not receiving antiviral drugs (No-NAs group) and 60 patients with CHB receiving TAF (TAF group). The Ethics Committee of Huashan Hospital, Fudan University approved this study (Ethics No: IRB no. KY2019-598). The study protocol conformed to the ethical principles of the Declaration of Helsinki, and the study was conducted following the approved study protocol. All participants provided written informed consent upon registration.

Inclusion criteria: (1) Patients with CHB (CHB of >6 months), excluding patients with hepatitis C virus, hepatitis D virus, and other hepatitis virus infections; and (2) aged 18–65 years. HBV DNA, HBeAg, HBsAg, HBsAb and HBeAb levels were detected during follow-up. Subjects enrolled in this study received TAF from 3 to 36 months. We divided CHB patients into two groups according to the HBeAg status of the subjects on the day of data collection. Phase 1 are HBeAg-negative patients and phase 2 are HBeAg-positive patients.

Exclusion criteria: (1) Patients infected within 3 months; (2) received antibiotics within 3 months; (3) received probiotics and probiotics within 3 months; (4) concomitant hypertension; (5) diabetes; (6) obesity or significantly low body weight; (7) obvious atherosclerosis; (8) chronic kidney disease; (9) history of gastrointestinal surgery; (10) inflammatory bowel disease; (11) irritable bowel syndrome; (12) malignant tumors; (13) autoimmune diseases; (14) Parkinson’s disease, Alzheimer’s disease, and stroke; (15) mental illness; (16) pregnant or lactating women; and (17) patients who had cirrhosis or decompensated liver disease.

## 3. Measurements

We collected data on medical records and sociodemographic characteristics of the study subjects. Serum alanine aminotransferase (ALT), aspartate aminotransferase (AST), and other blood parameters were measured using an automatic chemical analyzer. An electrochemiluminescence immunoassay was used to detect HBeAg and HBeAb. HBV DNA was quantified by real-time polymerase chain reaction.

### 3.1. Collection of stool samples and 16S rRNA sequencing

Participants’ stool samples were collected on the day of medical examination and immediately refrigerated at −80°C until analysis. The QIAamp PowerFecal DNA kit (Qiagen, DE) was used to extract DNA from fecal samples. The sequence of the V3-V4 region of the bacterial 16S rRNA gene was amplified from fecal DNA samples using forward primer 341F (5′-CCTACGGGNBGCASCAG-3′) and reverse primer 806R (5′-GGACTACNVGGGTWTCTAAT-3′). Sequencing was performed on the Illumina MiSeq platform (Illumina, San Diego, CA, USA) following the manufacturer’s instructions to produce 2 × 300 bp reads.

### 3.2. Bioinformatics analysis

Usearch (Version 11.0.667)^1^ was used to analyze sequencing data, and USEARCH-unoise3 to generate amplicon sequence variants (ASVs) tables (Edgar, 2016). Representative sequences of ASVs were aligned to the 16S V18 database using the RDP classifier^2^ for taxonomic classification. Species accumulation was analyzed using the vegan package, and Venn visualization was drawn using the ggvd package.^3^

### 3.3. Statistical analysis

Alpha diversity (ACE, Chaos1, Shannon, and Simpson) and beta diversity analyses based on the ASV table were performed using Vegan 2.5-7 (Oksanen et al., 2020). The adonis2 function in the vegan package was used for PERMANOVA analysis to evaluate the significance of differences between groups. The effect size (adonis2 *R*^2^) of metadata on microbiota was also calculated using the adonis2 function in the Vegan package with 999 permutations. PICRUSt2 (v2. 5. 1) analysis (Douglas et al., 2020) was performed using ASVs to infer the function of microbial communities. Linear Discriminant Analysis Effect Size (LEfSe) (Segata et al., 2011) was used to identify genera as well as metabolic pathways with differential abundance in different groups. All results were visualized using ggplot2 (Wickham, 2017). Significant correlations between microbial abundance and clinical properties were calculated by the corr.test function of the psych package (Revelle, 2022). All statistical analyses were performed on the R4.2 platform (R Core Team, 2013).

## 4. Sequence and data availability

The 16S sequencing raw reads for this study are available on NCBI SRA (accession number is NCBI SRA: PRJNA924551, and PRJNA778613). Metadata is available by mail to the authors.

## 5. Results

### 5.1. Basic characteristics of participants

Information on participants, including age, sex, body mass index (BMI), and blood chemistry parameters, is presented in Table 1. No significant differences were found in gender, age, and BMI among the four groups. HBV load, ALT, and AST were significantly higher in the No-NAs group, while prealbumin levels were significantly lower in the Phase 2 subgroup than in the Phase 1 subgroup. These measures did not significantly differ between the two subgroups of TAF. Additionally, other blood parameters demonstrated no significant differences.

**Table 1:** Demographic and clinical characteristics of study participants.

	No-NAs Phase 1 *N* = 40	No-NAs Phase 2 *N* = 55	TAF Phase 1 *N* = 20	TAF Phase 2 *N* = 40
Gender (female)	10 (25.0%)	26 (47.3%)	8 (40.0%)	12 (30.0%)
Age (year)	40.2 ± 7.8	35.9 ± 8.2	40.6 ± 10.0	35.3 ± 6.9
Body mass index	23.6 ± 3.2	22.7 ± 2.6	22.8 ± 2.4	21.8 ± 3.4
HBV DNA (log_10_)	3.4[1.9–4.3]^a^	7.6[6.5–7.9]^b^	0.0[0.0–0.0]^a^	0.4[0.0–2.7]^a^
Alkaline phosphatase (U/L)	84.5 ± 26.7	80.0 ± 23.6	76.4 ± 14.5	79.1 ± 18.0
r-Glutamyltransferase (U/L)	34.6 ± 35.1	35.4 ± 35.3	23.6 ± 10.7	25.3 ± 18.8
Alanine aminotransferase (U/L)	45.0 ± 28.7^a^	86.0 ± 102.9^b^	26.4 ± 11.7^a^	36.3 ± 31.1^a^
Aspartate aminotransferase (U/L)	31.9 ± 15.1^a^	48.2 ± 41.2^b^	25.1 ± 14.4^a^	24.1 ± 8.3^a^
Direct bilirubin (μmol/L)	4.2 ± 2.2	5.8 ± 11.9	3.9 ± 1.9	4.1 ± 1.6
Total bilirubin (μmol/L)	13.1 ± 6.3	12.1 ± 4.6	13.6 ± 6.7	13.7 ± 5.6
Total bile acids (μmol/L)	7.1 ± 2.5	7.8 ± 4.2	6.5 ± 1.1	6.2 ± 0.5
Prealbumin	233.5 ± 63.6^a^	194.5 ± 52.4^b^	248.9 ± 47.2^a^	249.0 ± 46.9^a^
Hemoglobin	151.4 ± 17.3	145.7 ± 16.8	139.5 ± 39.2	151.7 ± 15.2
Alpha-fetoprotein	3.1 ± 1.6	4.0 ± 5.8	3.0 ± 1.5	4.7 ± 7.4
Total protein	78.6 ± 4.6	77.3 ± 11.0	78.5 ± 6.5	78.5 ± 5.0
Albumin (g/L)	47.8 ± 3.4	47.3 ± 2.8	47.8 ± 2.8	48.1 ± 2.8
Albumin-globulin ratio	1.6 ± 0.2	1.5 ± 0.2	1.6 ± 0.2	1.6 ± 0.2
Globulin (g/L)	30.0 ± 6.3	33.6 ± 16.0	30.7 ± 4.7	30.4 ± 3.7
White cell (10^9/L)	6.0 ± 1.5	5.3 ± 1.6	5.3 ± 0.9	5.7 ± 1.4
Red cell (10^12/L)	5.0 ± 0.4	4.8 ± 0.5	10.0 ± 20.7	5.0 ± 0.4
Platelet count (10^9/L)	221.9 ± 54.4	217.2 ± 61.0	219.8 ± 93.8	221.2 ± 51.7
Urea (mmol/L)	5.2 ± 1.0	6.4 ± 12.1	5.4 ± 1.5	5.3 ± 1.1
Uric acid (mmol/L)	0.3 ± 0.1	0.5 ± 1.1	0.4 ± 0.1	0.3 ± 0.1
Creatinine (μmol/L)	68.3 ± 12.0	60.7 ± 13.9	65.5 ± 15.4	69.2 ± 13.0
Blood calcium (mmol/L)	2.3 ± 0.1	10.2 ± 25.2	2.3 ± 0.1	2.3 ± 0.1
Inorganic phosphorus (mmol/L)	1.0 ± 0.1	12.4 ± 42.3	1.0 ± 0.1	1.0 ± 0.1
eGFR (MDRD)	111.5 ± 15.8	121.3 ± 30.8	116.8 ± 24.2	118.4 ± 20.9
eGFR (EPI)	172.7 ± 189.4	454.5 ± 1670.7	109.9 ± 14.9	115.2 ± 10.7
β2-microglobulin	0.8 ± 0.9	0.4 ± 0.5	0.3 ± 0.1	0.4 ± 0.4

Categorical variables were analyzed by *χ*^2^ test, and continuous variables are presented as mean (standard deviation). ^a,b^Different letters indicate significant differences between groups.

### 5.2. Differences in gut microbiota between Phase 1 and Phase 2 in No-NAs group CHB patients

**Figure 1:**
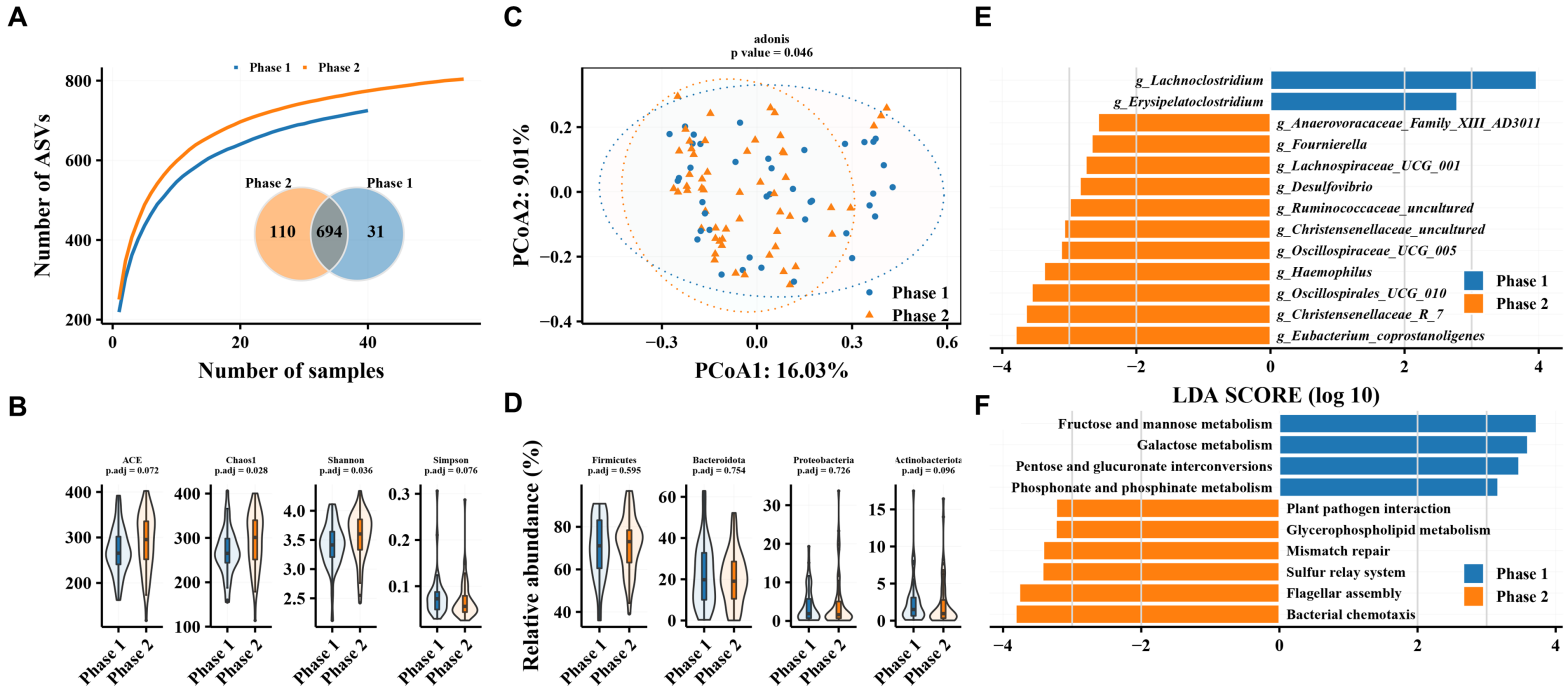
Characteristics of gut microbiota in patients with hepatitis B and differences in intestinal flora between patients in Phase 1 and Phase 2 subgroups. **(A)** Species accumulation curve and Venn diagram. **(B)** Alpha diversity analysis based on ASVs. **(C)** Principal coordinate analysis of β diversity of flora based on Bary-Curits distance (PCoA). **(D)** Differences in phylum levels of intestinal flora between patients with Phase 1 and Phase 2 chronic hepatitis B. **(E)** LEfSe analysis at genus level. **(F)** PICRUSt analysis. Phase 1 are HBeAg-negative patients and phase 2 are HBeAg-positive patients.

The results of species accumulation curves and the Venn plot (Figure 1A) revealed that 31 and 110 ASVs were independently present in Phase 1 and Phase 2 subgroups, respectively. This may be related to the larger sample size in the Phase 2 subgroup, but it indicates that the bacterial community in the Phase 2 subgroup tends to be heterogeneous. Alpha diversity analysis revealed similar results, with both gut microbiota richness (Chao1 index) and diversity (Shannon index) significantly increased in the Phase 2 subgroup. PCoA results revealed a significant difference in beta diversity in the gut microbiota between Phase 1 and Phase 2 subgroups (adonis2, value of *p* = 0.046). However, no significant difference was observed between Phase 1 and Phase 2 subgroups at the phylum level (Figure 1D). Firmicutes, Bacteroidetes, Proteobacteria, and Actinobacteria were the most abundant taxa, accounting for >97% of the total (Supplementary Figure S1A).

Phase 1 and Phase 2 subgroups revealed significant differences in intestinal bacterial composition. LEfSe analysis revealed that *Eubacterium_coprostanoligenes*, *Christensenellaceae_R_7*, *Oscillospirales_UCG_010*, and *Haemophilus* were enriched in the gut microbiota of Phase 2 subgroup compared with Phase 1 at the genus level, while the relative abundance of *Erysipelatoclostridium* and *Lachnoclostridium* was decreased. The Phase 1 subgroup was enriched in pathways related to glucose metabolism, such as fructose and mannose metabolism, galactose metabolism, and pentose and glucuronate interconversions as well as phosphonate and phosphinate metabolism, while the Phase 2 subgroup was enriched in pathways related to bacterial chemotaxis, flagellar metabolism, sulfur relay system, and plant pathogen assembly.

### 5.3. Differences in gut microbiota between Phase 1 and Phase 2 in CHB patients treated with TAF

**Figure 2:**
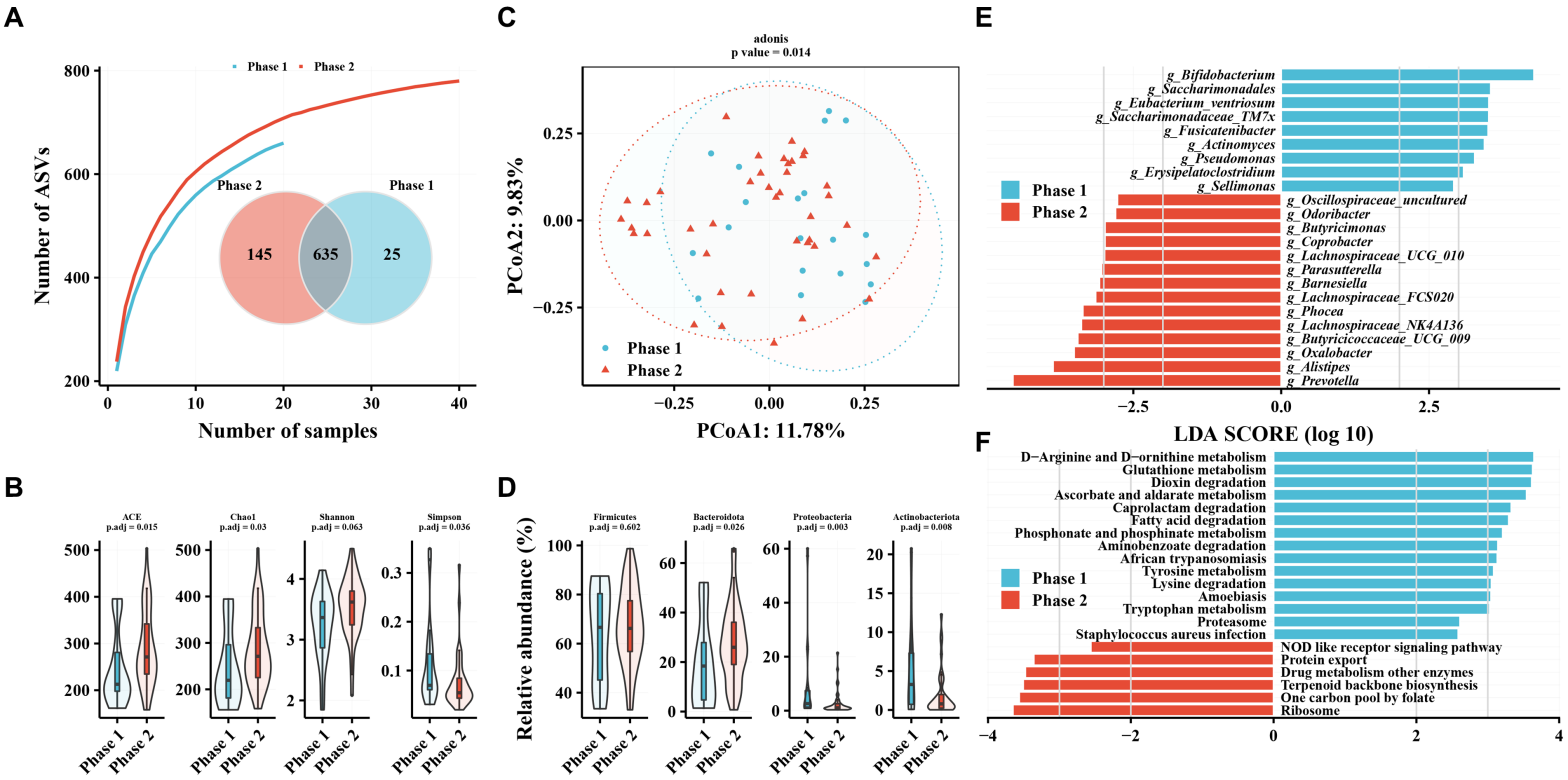
Characteristics of gut microbiota in patients with hepatitis B infection receiving TAF and differences in intestinal flora in Phase 1 and Phase 2 subgroups. **(A)** Species accumulation curve and Venn diagram. **(B)** Alpha diversity analysis based on ASV profile. **(C)** Principal coordinate analysis of β diversity of flora based on Bary-Curits distance (PCoA). **(D)** Differences in intestinal flora at phylum levels between patients with Phase 1 and Phase 2 chronic hepatitis B. **(E)** LEfSe analysis at genus level. **(F)** PICRUSt analysis. Phase 1 are HBeAg-negative patients and phase 2 are HBeAg-positive patients.

The TAF group had more independent ASVs in the Phase 2 subgroup (25 in Phase 1 and 145 in Phase 2), similar to the No-NAs group (Figure 2A). Alpha diversity analysis revealed significantly increased gut microbiota richness (ACE and Chao1 indices) and diversity (Simpson index) in the Phase 2 subgroup (Figure 2B). PCoA results revealed a significant difference in beta diversity in the gut microbiota between Phase 1 and Phase 2 subgroups (adonis2, value of *p* = 0.014) (Figure 2C). Firmicutes, Bacteroidetes, Proteobacteria, and Actinobacteria were the most abundant taxa in the gut microbiota of patients with CHB receiving TAF, as in the No-NAs group (Supplementary Figure S1B). However, significant differences were observed between the Phase 1 and Phase 2 subgroups at the phylum level, as shown by a significant increase in Bacteroidetes abundance and a significant decrease in Proteobacteria and Actinobacteria in the Phase 2 subgroup, unlike the No-NAs group (Figure 2D and Supplementary Figure S1B).

*Bifidobacterium*, *Saccharimonadales*, *Eubacterium_ventriosum*, and *Saccharimonadaceae_TM7x* were significantly lower in the Phase 2 subgroup compared with the Phase 1 subgroup at the genus level (Figure 2E). In contrast, several genera of the Phase 2 subgroup, such as *Prevotella*, *Alistipes*, *Oxalobacter*, *and Butyricicoccaceae_UCG_009*, were enriched compared with the Phase 1 subgroup (Figure 2E). Additionally, amino acid metabolism-related pathways were enriched in the Phase 1 subgroup, such as D-Arginine and D-ornithine metabolism, glutathione metabolism, tyrosine metabolism, lysine degradation, and tryptophan metabolism, in terms of metabolic pathways. Enrichment of related pathways, such as one carbon pool by folate, terpenoid backbone biosynthesis, and drug metabolism of other enzymes, were observed in the Phase 2 subgroup. Additionally, we observed enrichment of the phosphonate and phosphinate metabolism pathway in the Phase 1 subgroup in both patients unmedicated and TAF.

### 5.4. Effect of TAF on clinical parameters in patients with CHB

Table 2 shows the effect of TAF on clinical parameters in patients with CHB. The TAF group demonstrated a significantly decreased HBV load, AST, and ALT and a significant increase in prealbumin compared with the No-NAs group. No significant difference was found in uric acid, creatinine, blood calcium, inorganic phosphorus, eGFR, and β2-microglobulin concentrations between the two groups, indicating that TAF had little effect on renal function. Additionally, the TAF group had significantly lower urea levels than the No-NAs group.

**Table 2:** Demographic and clinical characteristics of subjects in No-NAs and TAF groups.

	No-NAs *N* = 95	TAF *N* = 60	*p* value
Medication time (mouth)		6.2 ± 5.1	
Gender (female)	36 (37.9%)	20 (33.3%)	0.686
Age (year)	36.5 ± 8.6	37.1 ± 8.4	0.776
Body mass index	23.1 ± 2.9	22.2 ± 3.1	0.075
HBV DNA (log10)	7.4 ± 7.5	3.2 ± 3.8	<0.001
Alkaline phosphatase (U/L)	81.7 ± 24.8	78.2 ± 16.9	0.432
r-Glutamyltransferase (U/L)	35.1 ± 35.0	24.8 ± 16.6	0.046
Alanine aminotransferase (U/L)	69.6 ± 83.9	33.2 ± 26.8	<0.001
Aspartate aminotransferase (U/L)	41.7 ± 34.2	24.4 ± 10.5	<0.001
Direct bilirubin (μmol/L)	5.2 ± 9.4	4.0 ± 1.7	0.671
Total bilirubin (μmol/L)	12.5 ± 5.3	13.7 ± 5.9	0.254
Total bile acids (μmol/L)	7.6 ± 3.6	6.3 ± 0.7	0.004
Prealbumin	209.5 ± 59.7	249.0 ± 46.5	<0.001
Hemoglobin	148.0 ± 17.1	148.0 ± 25.3	0.387
Alpha-fetoprotein	3.6 ± 4.6	4.2 ± 6.2	0.406
Total protein	77.8 ± 9.0	78.5 ± 5.4	0.998
Albumin (g/L)	47.5 ± 3.0	48.0 ± 2.8	0.318
Albumin-globulin ratio	1.5 ± 0.2	1.6 ± 0.2	0.213
Globulin (g/L)	32.2 ± 13.1	30.5 ± 4.0	0.307
White cell (10^9/L)	5.6 ± 1.6	5.6 ± 1.3	0.744
Red cell (10^12/L)	4.9 ± 0.5	6.5 ± 11.4	0.155
Platelet count (10^9/L)	219.1 ± 58.2	220.7 ± 66.4	0.647
Urea (mmol/L)	6.0 ± 10.0	5.3 ± 1.2	0.01
Uric acid (mmol/L)	0.4 ± 0.9	0.3 ± 0.1	0.112
Creatinine (μmol/L)	63.2 ± 13.7	68.1 ± 13.7	0.086
Blood calcium (mmol/L)	8.0 ± 21.3	2.3 ± 0.1	0.866
Inorganic phosphorus (mmol/L)	8.6 ± 34.5	1.0 ± 0.1	0.587
eGFR (MDRD)	119.1 ± 28.3	117.9 ± 21.6	0.465
eGFR (EPI)	387.8 ± 1461.1	113.6 ± 12.2	0.143
β2-microglobulin	0.6 ± 0.6	0.4 ± 0.3	0.829
Phase			0.356
Phase 1	40 (42.1%)	20 (33.3%)	
Phase 2	55 (57.9%)	40 (66.7%)	

No-NAs, patients with CHB not receiving antiviral drugs; TAF, tenofovir alafenamide.

### 5.5. Effect of TAF on gut microbiota in patients with CHB

**Figure 3:**
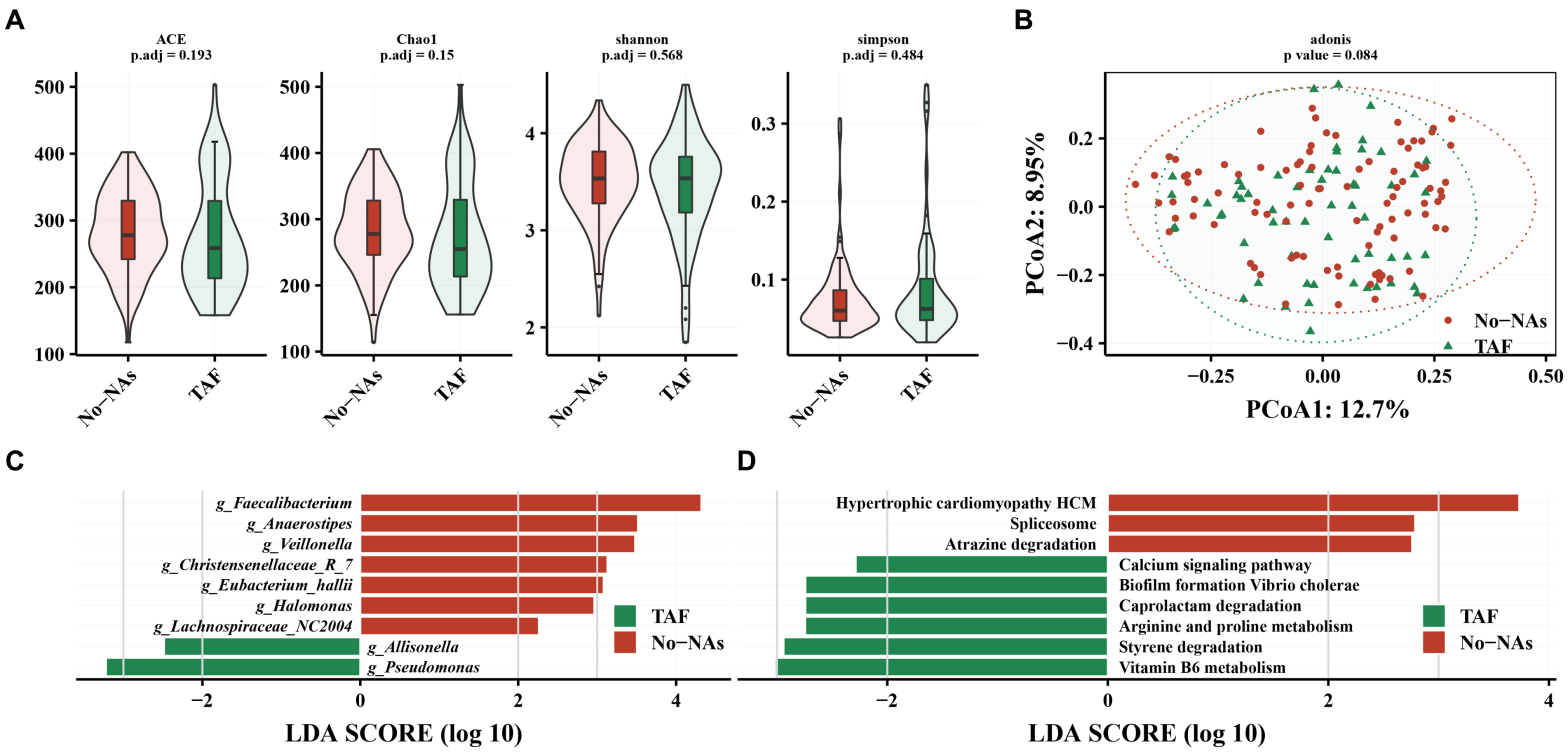
Effect of TAF on intestinal flora in patients with chronic hepatitis B. **(A)** Effect of TAF on alpha diversity of intestinal flora. **(B)** Principal coordinate analysis (PCoA) based on Bary-Curits distance to investigate the effect of TAF on beta diversity of intestinal flora. **(C)** LEfSe analysis at genus level. **(D)** PICRUSt analysis.

We compared the effect of TAF on gut microbiota. TAF demonstrated no significant effect on gut microbiota alpha diversity (Figure 3A) and beta diversity (Figure 3B) compared to patients with CHB in the No-NAs group. Additionally, differences were not observed in the relative abundance of gut microbiota at the phylum level between the TAF and No-NAs groups (Supplementary Figures S1C,D).

However, LEfSe analysis demonstrated a decreased *Faecalibacterium*, *Anaerostpes*, *Veillonella*, *Christensenellaceae_R_7*, *Eubacterium_hallii*, *Halomonas*, and *Lachnospiraceae_NC2004* and an increased relative abundance of *Pseudomonas* and *Allisonella* in the TAF group (Figure 3C). We performed a KEGG analysis to further understand the biological function of gut microbiota in patients receiving (TAF group) and patients not receiving (No-NAs group) TAF medications. Vitamin B6 metabolism, styrene degradation, arginine and proline metabolism, caprolactam degradation, biofilm formation vibrio cholerae, and calcium signaling pathway-related metabolic pathways were increased in the TAF group, while hypertrophic cardiomyopathy HCM, spliceosome e, and atrazine degradation metabolic pathways were decreased (Figure 3D).

### 5.6. Correlation analysis between intestinal flora and clinical indexes in patients with CHB

**Figure 4:**
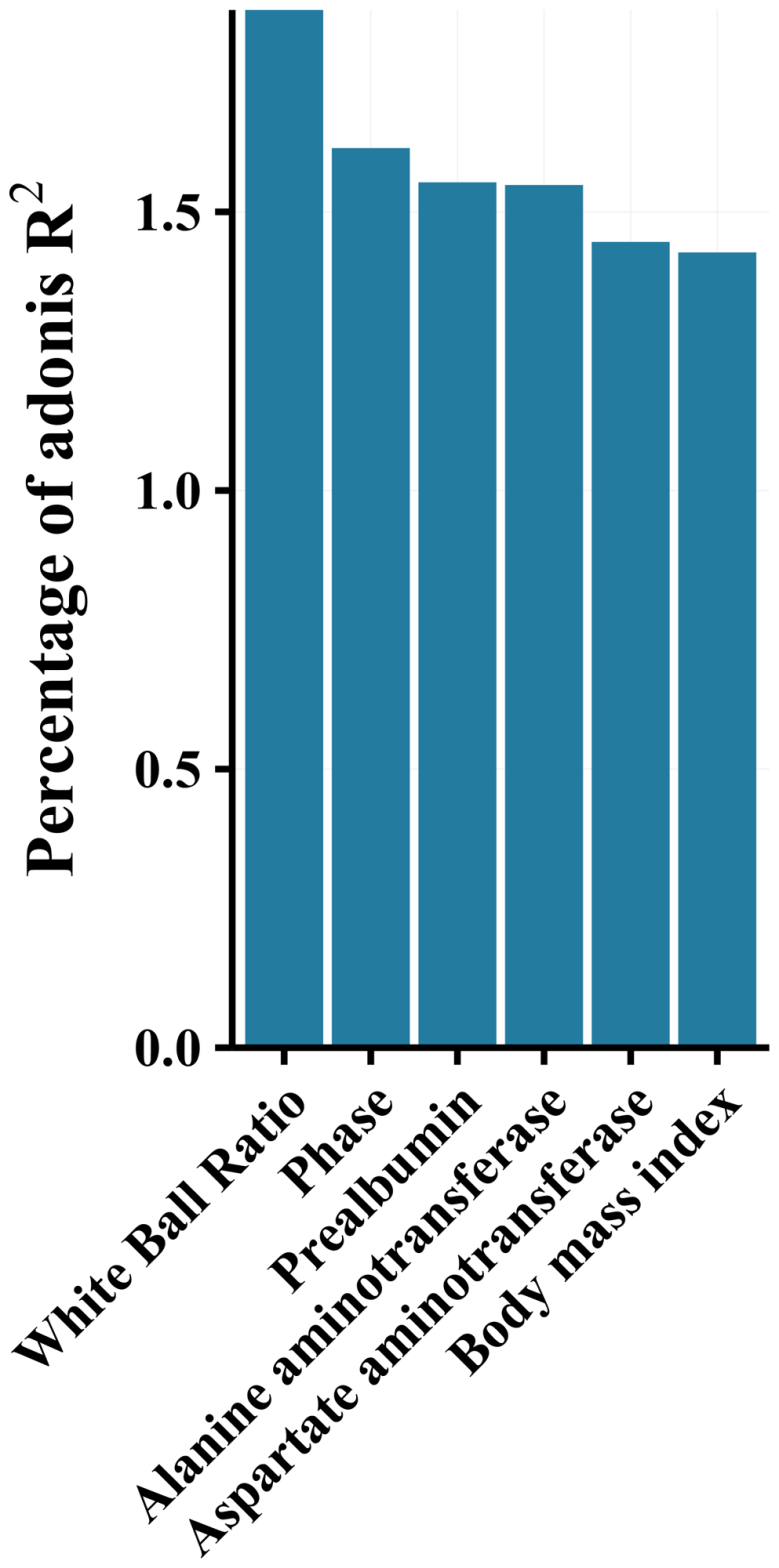
Effect size (adoniss *R*^2^) of metadata on microbiota was calculated using the adonis2 function in the vegan package with 999 permutations.

PERMANOVA analysis revealed a significant effect of disease stage on the community (*R*^2^ = 1.6, *p* < 0.001). Additionally, effect size analysis revealed that albumin-globulin ratio, prealbumin, ALT, AST, and BMI could explain approximately 1% of the variance of the gut microbiota, in addition to the presence or absence of HBeAg (Phase 1 and Phase 2) (*p* < 0.05, Figure 4). These results indicated that gut microbiota was significantly associated with albumin-globulin ratio, prealbumin, ALT, AST, and BMI.

**Figure 5:**
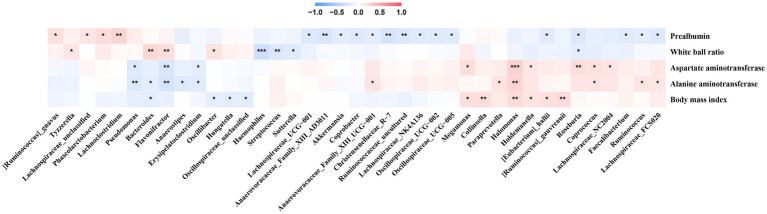
Correlation between clinical parameters and gut microbiota. *p-value smaller than 0.05, **p-value smaller than 0.01 and ***p-value smaller than 0.001.

We observed that *Erysipelatoclostridium* was negatively correlated with AST and ALT, and the bacterium was enriched in Phase 1 in the No-NAs and TAF groups (Figure 5). Additionally, *Lachnoclostridium* was positively correlated with prealbumin, and the bacterium was enriched in Phase 1 in the No-NAs group (Figure 1D). *Pseudomonas* was negatively correlated with AST and ALT (Figure 5), and the bacterium was enriched in Phase 1 in the TAF group (Figure 2D). Moreover, *Anaerovoracaceae_Family_XIII_AD3011*, *Ruminococcaceae_uncultured*, *Coprobacter*, and *Lachnospiraceae_NK4A136* were negatively correlated with prealbumin (Figure 5), and these bacteria were enriched in Phase 2 of the No-NAs or TAF groups (Figures 1D, 2D). *Haemophilus* enriched in Phase 2 was negatively correlated with the albumin-globulin ratio in the No-NAs group.

*Halomonas* was positively correlated with AST, ALT, and BMI (Figure 5), and the bacterium was enriched in the No-NAs group (Figure 3C), indicating that TAF could reduce the abundance of the bacterium. Additionally, *Faecalibacterium* and *Lachnospiraceae_NC2004*, which were negatively correlated with prealbumin, were also enriched in the No-NAs group (Figure 3C), and particularly *Lachnospiraceae FCS020* was enriched in Phase 2 in the TAF group (Figure 2E), and the bacterium was positively correlated with ALT (Figure 5).

## 6. Discussion

This study investigated differences between gut microbiota in patients with HBeAg-positive and -negative CHB. The results revealed significantly elevated richness and diversity of gut microbiota in HBeAg-positive patients, showing greater heterogeneity. Additionally, the Phase 2 subgroup demonstrated a low abundance of pathways associated with glucose or amino acid metabolism. Moreover, patients with HBeAg-negative and -positive chronic HBV infection have a low viral load, high HBsAg clearance, good long-term prognosis, and low risk of further progression to cirrhosis and HCC (Invernizzi et al., 2016). These results indicate that gut microbiota is associated with HBV infection development. The gut microbiomes of HBV-infected individuals are highly diverse, and to our knowledge, this is the first time that differences in gut microbiota have been evaluated based on HBeAg status in patients with CHB.

No significant difference was found in blood calcium, inorganic phosphorus, β2-microglobulin, and eGFR contents in patients in the TAF group compared with the No-NAs group (Table 2), indicating that TAF had little effect on renal function. This is consistent with literature reports that TAF recipients have a higher rate of proximal renal function preservation and less phosphate loss from the proximal renal tubules (Sax et al., 2014, 2015; Wohl et al., 2016).

Additionally, we evaluated for the first time the effect of TAF on gut microbiota in patients with CHB. The results revealed that TAF resulted in visible, but not significant changes in gut microbiota in patients with CHB compared with the No-NAs group. Significant effects of entecavir on gut microbiota have been reported in patients with CHB (Lu et al., 2021). This study revealed no significant changes in gut microbiota, which may be related to the low use of TAF and low plasma pK exposure. In general, TAF (25 mg) was used at a lower dose than TDF (245 mg), resulting in a 90% reduction in TAF plasma concentrations (Lee et al., 2005; Sax et al., 2014). Further, the variable length of medication in the TAF group (1–36 months, mean = 5.5 months), brings some heterogeneity. Intestinal microbial changes are one of the causes of systemic immune activation caused by chronic HBV infection. Numerous studies on the gut-liver axis have the important role of gut microbiota in CHB development (Chou et al., 2015; Zhu et al., 2019). Additionally, we observed that TAF caused changes in the metabolic pathways of gut microbiota. Vitamin B6 metabolism, arginine and proline metabolism, caprolactam degradation, and calcium signaling pathway-related metabolic pathways increased in abundance in the TFA group (Figure 3D). Gut microbe translocation and its products have been suggested to exacerbate clinical symptoms in patients with CHB virus infection (Tsiaoussis et al., 2015; Kang and Cai, 2017). However, reports in this area are limited. Therefore, future further clinical trials to investigate the effects of TAF on gut microbiota and metabolism are beneficial to understand the relationship between gut microbiota and CHB, as well as for better treatment modality development. Bile acids (BAs) influence the structure and function of the gut microbiota, whereas the metabolic capacity of the gut microbiota and external factors such as antibiotics and diet may influence the composition of Bas (Collins et al., 2023). Bidirectional interactions between the gut microbiota and metabolome are becoming increasingly important for diseases such as metabolic and tumor diseases. Recent studies have shown that not only is the gut microbiota altered in CHB patients, but also the proportion of conjugated BAs and primary BAs is significantly increased in CHB patients (Sun et al., 2021). Bao et al. (2023) demonstrated alterations and interactions in the gut microbiome and BA during enterohepatic circulation in patients with acute-chronic liver failure and sub-massive liver necrosis. Thus, modulation of the gut microbiota could become an important tool to improve the response to CHB/HCC immunotherapy (Shen et al., 2022).

This study covers patients with CHB in the No-NAs group and patients with CHB receiving TAF, as well as a group study of patients with HBeAg-positive and -negative CHB, to reveal the characteristics of the gut microbiota in patients with different stages of CHB virus, which may help improve the therapeutic effect in patients with CHB by intervening the gut microbiota in the future. However, our study has several limitations. First, patients in the No-NAs and TAF groups were not the same, so we could not conclude the effect of TAF on gut microbiota by self-control. Second, our sample size was not large enough, and we just observed the potential of TAF to influence gut microbiota. Therefore, the effect of TAF on gut microbiota should be evaluated through a prospective self-controlled trial with a large sample in the future. Additionally, the future treatment of patients with CHB by probiotics combined with TAF may bring better benefits to patients and is also a topic worthy of further study in the future, considering the important role of gut microbes in liver disease development.

## 7. Conclusion

In conclusion, we investigated gut microbiota alterations in HBeAg -positive and -negative subjects from patients with CHB and the effect of TAF on gut microbiota. Beneficial bacteria, such as *Lachnoclostridium*, *Erysipelatoclostridium*, and *Bifidobacterium*, were reduced in the HBeAg -positive group (Phase 2). The abundance of pathways related to glucose and amino acid metabolism decreased in the HBeAg -positive group (Phase 2) on metabolic pathways. Additionally, clinical features and gut microbiota demonstrated correlations, particularly with changes in leukocyte ratio, prealbumin, AST, and ALT levels. Alternatively, TAF intervention caused visible but insignificant changes in gut microbiota compared to the No-NAs group.

## Data availability statement

The data presented in the study are deposited in the NCBI repository, accession number SRA: PRJNA924551, and PRJNA778613.

## Ethics statement

The studies involving humans were approved by the Ethics Committee of Huashan Hospital, Fudan University approved this study (Ethics No: IRB no. KY2019-598). The studies were conducted in accordance with the local legislation and institutional requirements. The participants provided their written informed consent to participate in this study. Written informed consent was obtained from the individual(s) for the publication of any potentially identifiable images or data included in this article.

## Author contributions

LJ participated in designing of the study, collected the samples, and wrote the manuscript. GJ sub packaged the fecal specimens and collected the clinical data. ZH, LX, LL, and CB supplemented and updated the literature. RH and LC performed the 16S rRNA gene sequencing and the bioinformatics analysis. ZJ explained the informed consent form and designed the experiments. LH and WB revised the manuscript. All authors read through and approved the final manuscript.

## Footnotes

^1^ http://www.drive5.com/usearch/

^2^ https://www.drive5.com/usearch/manual/sintax_downloads.html

^3^ https://github.com/csdaw/ggvd
