# Corrigendum: Difference of gut microbiota between patients with negative and positive HBeAg in chronic hepatitis B and the effect of tenofovir alafenamide on intestinal flora

**DOI:** 10.3389/fmicb.2023.1322004

**Published:** 2023-11-06

**Authors:** Jianfei Long, Jingru Gong, Han Zhu, Xiaolin Liu, Ling Li, Bicui Chen, Hongyan Ren, Chao Liu, Huiping Lu, Jiming Zhang, Bin Wang

**Affiliations:** ^1^Department of Pharmacy, Huashan Hospital, Fudan University, Shanghai, China; ^2^Department of Pharmacy, Shanghai Pudong Hospital, Fudan University Pudong Medical Center, Shanghai, China; ^3^Department of Pharmacy, Jing'an District Central Hospital, Fudan University, Shanghai, China; ^4^Shanghai Mobio Biomedical Technology Co., Ltd., Shanghai, China; ^5^Department of Infectious Diseases, Shanghai Key Laboratory of Infectious Diseases and Biosafety Emergency Response, National Medical Center for Infectious Diseases, Huashan Hospital, Fudan University, Shanghai, China; ^6^Shanghai Institute of Infectious Diseases and Biosecurity, Key Laboratory of Medical Molecular Virology (MOE/MOH), Shanghai Medical College, Fudan University, Shanghai, China; ^7^Department of Infectious Diseases, Jing'An Branch of Huashan Hospital, Fudan University, Shanghai, China

**Keywords:** hepatitis B virus, HBeAg, HBsAg, tenofovir alafenamide, gut microbiota

In the published article, there was an error in Figure 4. The horizontal axis previously stated, “White ball ratio” which should be corrected as follows: “Albumin-globulin ratio”. The corrected Figure appears below.

**Figure 4:**
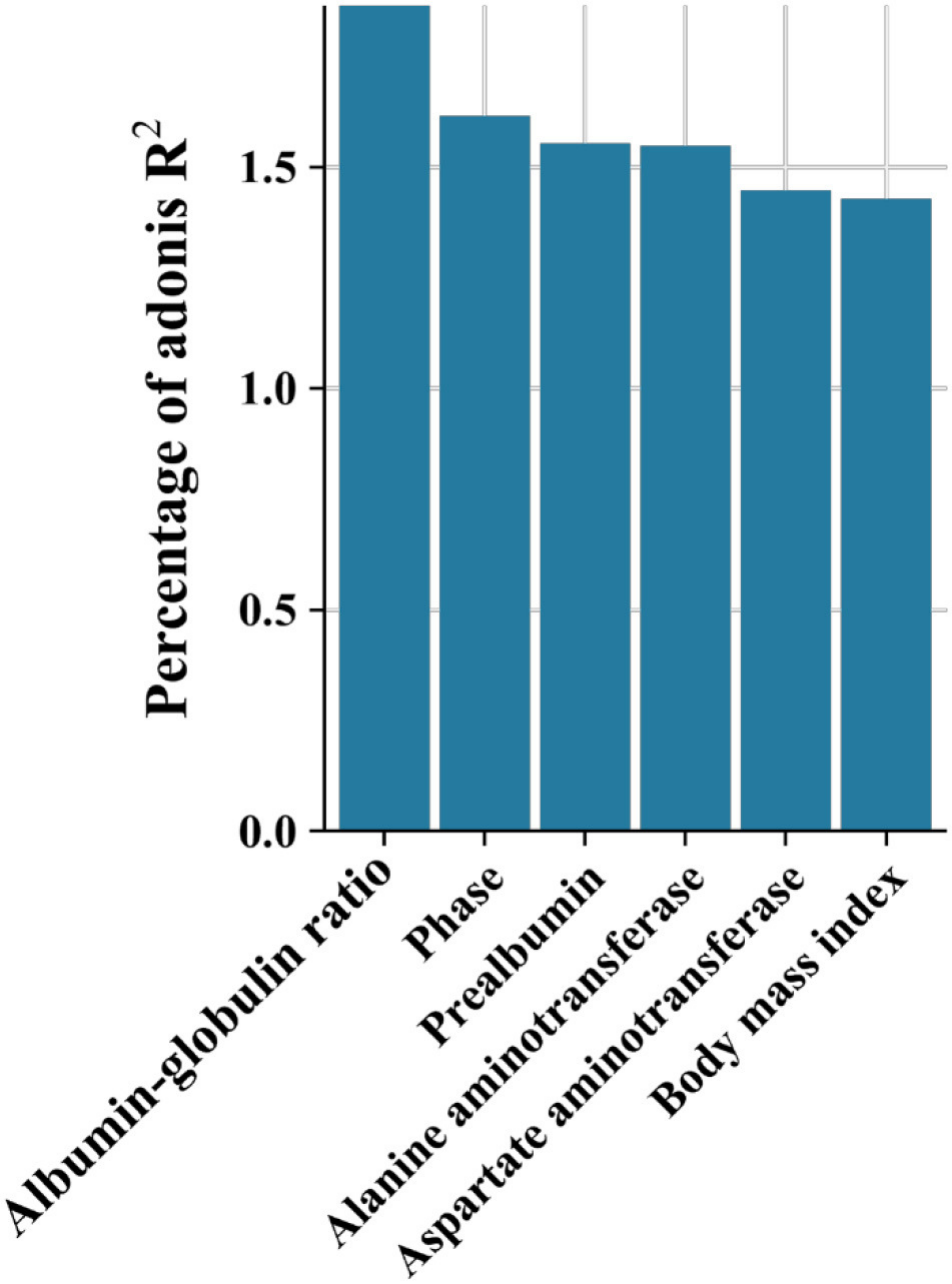
Effect size (adoniss *R*^2^) of metadata on microbiota was calculated using the adonis2 function in the vegan package with 999 permutations.

In the published article, there was an error in the **Results** subsection 5.2, page 3. “Differences in gut microbiota between Phase 1 and Phase 2 in No-NAs group CHB patients”.

“The results of species accumulation curves and the Venn plot (Figure 1A) revealed that 31 and 118 ASVs were independently present in Phase 1 and Phase 2 subgroups, respectively.”

The corrected sentence appears below:

“The results of species accumulation curves and the Venn plot (Figure 1A) revealed that 31 and 110 ASVs were independently present in Phase 1 and Phase 2 subgroups, respectively.”

The authors apologize for this error and state that this does not change the scientific conclusions of the article in any way. The original article has been updated.

