# Supplementary figures and images for: Difference of gut microbiota between patients with negative and positive HBeAg in chronic hepatitis B and the effect of tenofovir alafenamide on intestinal flora
Source: Front Microbiol. 2023 Sep 20;14:1232180. doi: 10.3389/fmicb.2023.1232180 (PMC10548823; doi:10.3389/fmicb.2023.1232180)

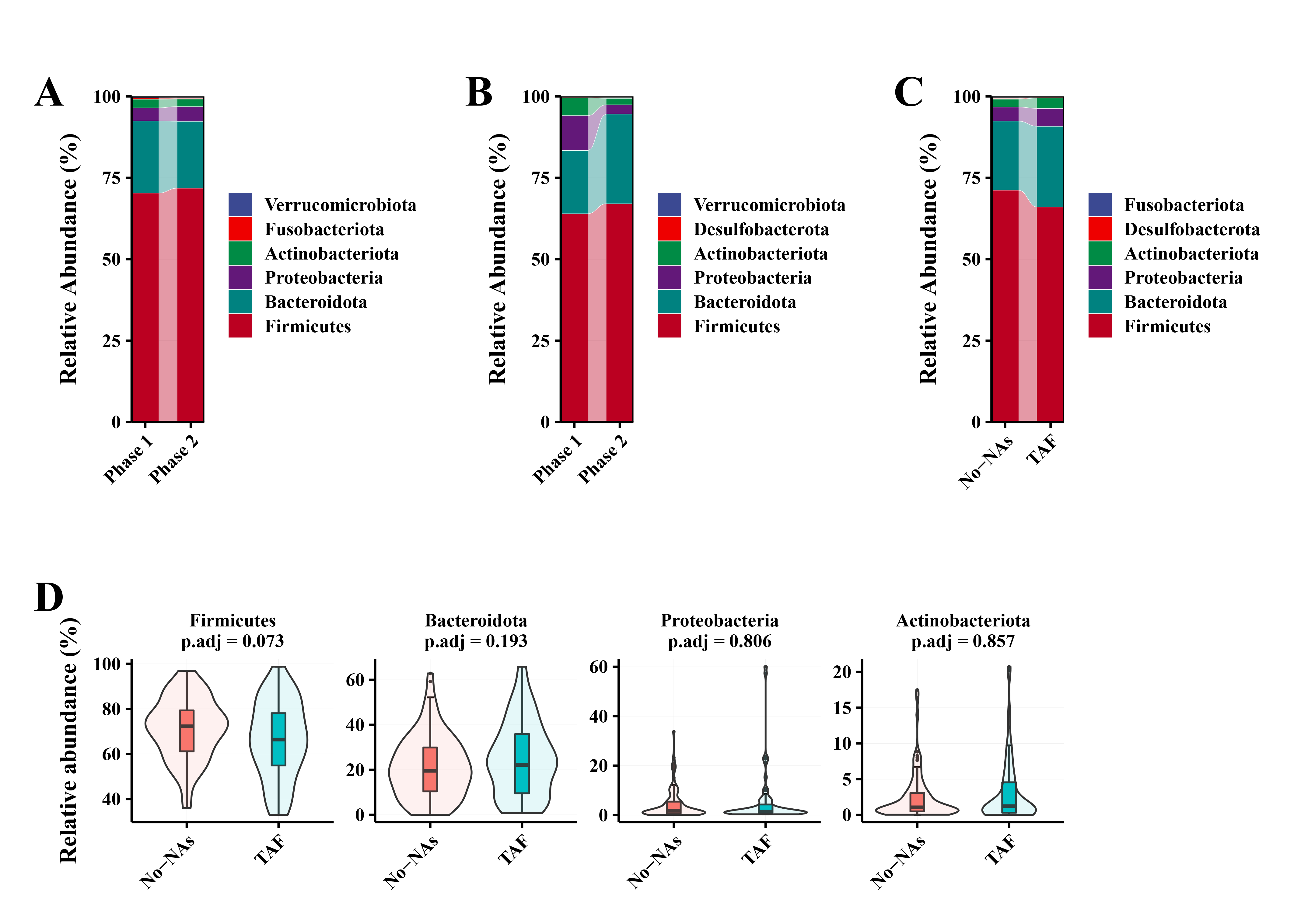

Supplement: Supplementary file 1 [file Image_1.tif]
